# Comprehensive mapping of SIVmac239 envelope antigenic determinants recognized by specific polyclonal antibodies in vaccinated and/or infected macaques

**DOI:** 10.1186/1742-4690-9-S2-P47

**Published:** 2012-09-13

**Authors:** J Guo, T Zuo, L Zhang, Z Chen

**Affiliations:** 1 AIDS Institute, LKS Faculty of Medicine, The University of Hong Kong, Hong Kong, China; 2 AIDS Research Center, School of Medicine, Tsinghua University, Beijing, China

## Background

The RV144 trial has demonstrated a modest level of protection against HIV infection, which was partially related to binding antibodies targeting V2 loop. Along with several broad HIV-neutralizing antibodies identified recently, it is of great importance to understand the major differences between vaccination and infection in eliciting humoral immune responses.

## Methods

We adopted a robust mapping technique for a quantitative measurement of antigenic determinants of entire SIVmac239 envelope glycoprotein displayed on the surface of the yeast as a combinatorial antigen library. Positive yeast clones recognized by the immunized and/or infected serum were identified and obtained by FACS followed by sequencing and structural analysis.

## Results

The finding of reactive determinants ranges from 30 to 240 amino acids, which allows some conformational determinants being evaluated as a major technical improvement. The antigen profile of the two types of vaccine-induced sera before viral challenge shared two dominant domains that concentrated at the V1V2 stem of gp120 and the ecto-domain of gp41. Interestingly, using a FACS-based antibody binding assay, there were no significant differences of titer and MFI of anti-V1V2 antibodies between the two vaccination groups, suggested a minimal role of these antibodies in controlling viral replication post SIVmac239 challenge. A major distinct domain, however, was identified near the V3 loop and the main CD4 binding region which was significantly recognized by the immune serum of the effective vaccination regimen but not of the non-effective vaccination regimen or natural SIV infection. Unexpectedly, the anti-V1/V2 antibody responses were shifted to the ecto-gp41 region when infected macaques developed AIDS.

## Conclusion

We present a comprehensive analysis of antigenic determinants recognized by specific antibody responses generated by vaccination and/or SIVmac239 infection. Our findings have significant implications to help understanding the humoral immunity against neutralization-resistant SIVmac239 infection and simian AIDS, and to guide rational vaccine immunogen identification and design.

